# Vagal modulation in pediatric disorders of gut–brain interaction: the role of 24 h heart rate variability

**DOI:** 10.1007/s10286-026-01186-4

**Published:** 2026-02-05

**Authors:** Gisela Chelimsky, Julian F. Thayer, DeWayne P. Williams, Le Kang, Thomas Chelimsky

**Affiliations:** 1 Department of Pediatrics, Virginia Commonwealth University, Richmond, VA USA; 2 School of Social Ecology, University of California Irvine, Irvine, CA USA; 3 Department of Biostatistics, Virginia Commonwealth University, Richmond, VA USA; 4 Department of Neurology, Virginia Commonwealth University, Richmond, VA USA

**Keywords:** Disorders of brain gut interaction, Heart rate variability, Vagal modulation, Pediatrics

## Abstract

**Background:**

Disorders of gut–brain interaction (DGBI) are common in pediatrics. Though the name clearly implies a neural contribution, the role of the autonomic nervous system remains unclear. Heart rate in healthy subjects (HC) follows a circadian pattern with dipping during the night with increased high frequency (hf) heart rate variability (HRV) and root mean square of successive differences (RMSSD). Our hypothesis was that reduced vagal modulation in adolescents with DGBI is associated with blunted rise in nocturnal vagal modulation.

**Methods:**

An institutional review board (IRB) approved this study, which included children aged 12–18 years with a DGBI and carefully screened HC. All subjects underwent 24 h HRV recording. The following questionnaires/tools were included: Pain Catastrophizing Scale for Children (PCS-C), Pain Catastrophizing Scale for Parents (PCS-P), Revised Child Anxiety and Depression Scale (RCADS), Trauma Symptom Checklist for Children (TSCC), and Functional Disability Inventory (FDI).

**Results:**

In total, 12 HC and 15 participants with DGBI participated (female individuals with DGBI versus HC: 93% versus 58%, *p* = 0.08). There was no age difference (median [range] HC 16.6 years [13.4, 18.2]; DGBI 16.8 years [13.8, 18.7], *p* = 0.92). The 24 h, daytime and nighttime HRV demonstrated that RMSSD, low-frequency (lf) HRV, and high-frequency (hf) HRV were lower in the DGBI group (*p* < 0.001). A nocturnal rise in RMSSD was present in the DGBI group, but less so in the HC group (*p* = 0.021). Higher catastrophizing correlated with lower nocturnal RMSSD (PCS-C correlation coefficient [CC]: −0.46), depression (RCADS depression CC: −0.51), and post-traumatic stress disorder (PTSD; TSCC PTSD CC: −0.58).

**Conclusions:**

HRV is reduced in patients with DGBI with a blunted rise in nocturnal RMSSD at night. As expected, vagal modulation is inversely correlated with catastrophizing, depression, PTDS, and FDI, with nocturnal HRV showing generally better correlations than daytime HRV.

## Introduction

Disorders of gut–brain interaction (DGBI) are common in pediatrics, affecting between 10% and 23% of the population [1]. Though the very name clearly implies a neural contribution to these disorders, the role of the autonomic nervous system remains unclear. We previously reported that adolescents with DGBI have decreased vagal modulation during a supine and standing challenge compared with healthy control subjects [2]. Heart rate variability (HRV), particularly the high frequency portion (hf) is a clear reflection of cardio-vagal modulation (Laborde S. 2017) and therefore reflects vagal function.

The regulation of visceral function typically requires an interaction between the sympathetic and parasympathetic nervous systems. When the activation of one branch produces the withdrawal of the other branch, this is termed “sympatho-vagal balance.” In other settings, both branches can be activated or withdrawn together [3]. In the analysis of HRV, very low frequency (VLF) is a marker of humoral and hormonal changes and low frequency (lF) is a marker of baroreceptor response; therefore, both sympathetic and parasympathetic responses and high frequency (hf) are nearly pure markers of vagal modulation [3–5].

Heart rate in healthy subjects follows a circadian pattern with dipping during the night with increased hf HRV and root mean square of successive differences (RMSSD). This is more pronounced in younger adults aged 20–50 years than in older adults > 50 years of age [6]. “Nocturnal dipping “ is considered to be cardiovascularly restorative [7]. Non-dipping has been defined by some authors as a decrease at night of the heart rate of less than 10% of the daytime values. Blunted dipping in heart rate in the general population is associated with preclinical cardiovascular disease, perhaps reflecting increased nocturnal sympathetic activity [8].

To the best of our knowledge, this is the first report of 24 h HRV in pediatric patients with DGBI enrolled from a neurogastroenterology clinic.

This study aimed to: (1) corroborate our previous findings of decreased hf HRV and therefore vagal modulation in adolescents with DGBI by analyzing 24 h HRV rather than evaluating an isolated 30–40 min time period; (2) better understand the alterations in circadian rhythm that these patients may experience through nocturnal HRV; (3) evaluate the relationship between ability to function measured by the Functional Disability Inventory (FDI) and hf HRV; (4) determine whether anxiety, depression, catastrophizing, and history of trauma correlate inversely with hf HRV.

## Methods

This was an institutional review board-approved study performed at Children’s Wisconsin in Milwaukee between April 2015 and March 2018. We enrolled adolescents and children aged 12–18 years with a DGBI who were seen in the pediatric neurogastroenterology and autonomic clinic. We included subjects with DGBI on the basis of ROME III criteria, including functional dyspepsia, irritable bowel syndrome (IBS), functional abdominal pain, and functional abdominal pain syndrome. We did not include patients with only functional constipation or diarrhea or only rumination. Many patients with DGBI had been under treatment for some time and had improved their function and other comorbidities such as depression and anxiety to the point that their levels were similar to the healthy subjects.

Carefully screened healthy control subjects were excluded if they had any chronic pain syndrome, DGBI, fibromyalgia assessed by history and tender point examination as described by the American College of Rheumatology, chronic sleep complaints, chronic fatigue, dizziness, or history of > 3 syncopal episodes in their life. Healthy controls were enrolled using flyers placed at the Children’s Wisconsin hospital and word of mouth. We did not enroll siblings of subjects with DGBI as healthy controls given that often these disorders run in families.

Participants and legal guardians came to the pediatric research unit. While there, after consent was obtained, the children, and when appropriate, the parent, answered several questionnaires, including the Pain Catastrophizing Scale for Children (PCS-C) and PCS for Parents (PCS-P), Revised Child Anxiety and Depression Scale (RCADS), Trauma Symptom Checklist for Children (TSCC), and Functional Disability Inventory (FDI). The TSCC is a 20-item questionnaire that has two subscales, general trauma (12 items) and sexual concern (8 items). The PCS is widely used to measure pain catastrophizing. The FDI is widely used in pediatric gastroenterology to measure function. The RCADS is a 25-item questionnaire that assesses depression and anxiety. After completing the questionnaires, a Faros device (Bittium, Finland) that records electrocardiogram (ECG) signals was attached to the participants’ chest and sent home with the individual to record ECG for the next 24 h. A diary of sleep and wake times was also completed.

The acquired raw ECG signal obtained from the Faros device was then processed for artifact removal and analyzed with Kubios 2.2 for high-frequency (hf) and low-frequency (lf) heart rate variability (HRV) using Kubios’ autoregressive spectral analysis software. Absolute values were log-transformed owing to skewness. Daytime and nighttime recordings were distinguished primarily on the basis of the sleep log that patients presented and confirmed using the Faros instrument’s actigraphy feature to register position (supine versus standing). Nocturnal time period was taken as starting 1 h after patient-recorded sleep time and ending 1 h prior to recorded awakening, to maximize the chance of a true sleep record.

The continuous measures were summarized using mean and standard deviation, or median with range. The categorical measures were summarized using frequencies and percentages. Comparisons between HC and DGBI subjects were performed using the Wilcoxon rank-sum test for continuous data, and chi-squared test for categorical data, respectively. Pearson’s correlation coefficients were estimated to quantify the degree of association between various measures. A Fisher *z*-transformation was used for correlation coefficients to yield a variable whose distribution is approximately normally distributed, and then Fisher’s *z*-test was used to compare the correlation coefficients between HC and DGBI. All statistical analyses were conducted using R 4.4.2 (R Foundation for Statistical Computing, Vienna, Austria). 

## Results

We enrolled 12 HC and 15 individuals with DGBI. The DGBI group had a preponderance of female participants (93% versus 58%, *p* = 0.08), but there was no difference in age (median [range]: HC 16.6 years [13.4, 18.2]; DGBI 16.8 years [13.8, 18.7], *p* = 0.92).

### Results of HRV parameters during nighttime and daytime

The 24 h HRV showed that RMSSD, log of low frequency (lf) HRV, and log of hf HRV were significantly lower in the DGBI group (*p* < 0.001). When dividing the same parameters by daytime and nighttime, the HRV parameters were also significantly lower in the DGBI group (Table 1). While the DGBI group still had significant nocturnal increase in RMSSD, it was less than that seen in the HC group (*p* = 0.021).

**Table 1:** Heart rate variability values in healthy controls and DGBI group

	Healthy subjects		DGBI		*p*-Value
	Mean (SD)	Median (min, max)	Mean (SD)	Median (min, max)	
24 h HRV RMSSD (ms)	54.6 (11.6)	56.9 (33.6, 71.7)	29.3 (10.7)	28.6 (11.3, 50.1)	< 0.001
24 h hf HRV (log)	6.88 (0.4)	6.91 (6.05, 7.47)	5.58 (1.19)	5.80 (2.82, 7.75)	< 0.001
24 h lf HRV (log)	7.66 (0.4)	7.71 (6.90, 8.22)	6.15 (1.87)	6.66 (0, 7.31)	< 0.001
Daytime RMSSD (ms)	39.0 (9.4)	38.8 (24.9, 59.9)	20.9 (7.81)	21.8 (7.81, 30.9)	< 0.001
Daytime hf HRV (log)	5.61 (0.9)	5.87 (4.07, 7.16)	4.57 (1.11)	4.74 (2.47, 5.94)	0.007
Daytime lf HRV (log)	7.45 (0.4)	7.52 (6.69, 7.93)	6.47 (0.815)	6.64 (4.54, 7.66)	0.001
Nighttime RMSSD	73.6 (21.9)	78.4 (33.6, 106)	36.8 (18.8)	38.3 (8.77, 70)	< 0.001
Nighttime hf HRV (log)	7.40 (0.7)	7.54 (6.17, 8.26)	5.93 (1.25)	5.72 (3.6, 7.6)	0.001
Nighttime lf HRV (log)	7.65 (0.51)	7.68 (6.64, 8.46)	6.14 (1.89)	6.60 (0, 7.91)	< 0.001

### Psychological measures

The psychological measures showed no significant differences between the two groups.

### Correlation between HRV and psychological measures by groups

In the 24 h HRV analysis for the DGBI group, the inverse correlation of RMSSD with catastrophizing (PCS-C: −0.46), depression (RCADS depression: −0.51), and post-traumatic stress disorder (PTSD; TSCC PTSD: −0.58) was not surprising. Neither was it surprising that catastrophizing in the parent also correlated inversely with hf HRV in the adolescent in the 24 h analysis (−0.66), which was less inversely correlated with daytime hf HRV (−0.23) and more with nighttime hf HRV (−0.76) and RMSSD (−0.47) (Table 3). The 24 h RMSSD (−0.46) and the hf HRV (−0.49) were also inversely correlated with the FDI in the DGBI group, which also showed a significant difference when compared with the healthy control group (*p* = 0.04). Interestingly, the daytime hf HRV and RMSSD inverse correlation with FDI were less than the 24 h HRV, while the nighttime was similar (Tables 2 and 3).

**Table 2:** Psychological measures in healthy control and DGBI groups

	Healthy subjects		DGBI		*p*-Value
	Mean (SD)	Median (min, max)	Mean (SD)	Median (min, max)	
PCS-C total score (0–52)	14.8 (9.71)	13.5 [2.00, 36.0]	17.4 (8.56)	14.0 [9.00, 34.0]	Not significant (NS)
PCS-P (0–52)	20.4 (10.7)	16.5 [8.00, 39.0]	21.3 (11.4)	20.5 [7.00, 42.0]	NS
RCADS total score (0–52)	42.8 (9.25)	42.0 [32.0, 66.0]	40.9 (6.33)	39.0 [33.0, 54.0]	NS
RCADS raw depression	8.08 (5.20)	8.50 [0, 19.0]	8.31 (3.86)	9.00 [2.00, 15.0]	NS
RCADS raw total anxiety	19.9 (12.4)	19.5 [2.00, 49.0]	17.1 (6.47)	16.0 [9.00, 29.0]	NS
TSCC *T*-score post-traumatic stress	45.0 (8.40)	41.5 [36.0, 65.0]	42.1 (6.32)	41.0 [35.0, 57.0]	NS
FDI	14.8 (15.0)	6.00 [0, 43.0]	16.1 (13.6)	17.0 [0, 40.0]	NS

**Table 3:** Correlation of 24 h HRV data with psychological measures in healthy control and DGBI groups

	RMSSD			lf HRV			hf HRV			lf/hf		
	HC	DGBI	*p*-Value	HC	DGBI	*p*-Value	HC	DGBI	*p*-Value	HC	DGBI	*p*-Value
PCS-C	−0.29	−0.46	0.64	−0.37	0.07	0.30	−0.24	−0.57	0.36	−0.19	0.78	0.01
PCS-P	0.18	−0.08	0.56	−0.15	−0.66	0.14	0.10	−0.12	0.62	−0.39	0.10	NS
RCADS d	−0.05	−0.51	0.24	−0.24	−0.05	0.66	0.03	−0.56	0.13	−0.44	0.61	0.01
RCADS a	−0.33	−0.15	0.66	−0.39	0.02	0.33	−0.25	−0.21	0.93	−0.24	0.47	NS
RCADS a and d	−0.28	−0.4	0.77	−0.37	0.04	0.32	−0.19	−0.47	0.47	−0.29	0.67	0.01
TSCC PTSD	−0.21	−0.58	0.31	−0.13	0.28	0.34	−0.02	−0.68	0.07	−0.20	0.84	0.01
FDI	0.38	−0.46	0.04	0.28	−0.21	0.25	0.34	−0.49	0.04	−0.14	0.37	NS

*RCADS d* RCADS depression, *RCADS a* RCADS anxiety, *RCADS a and d* RCADS total anxiety and depression, *HC* healthy control, *DGBI* disorders of gut–brain interaction, *lf HRV* low-frequency HRV, *hf HRV* high-frequency HRV

### Correlations within psychological measures across groups

PCS-P and PCS-C correlated mildly with a coefficient of 0.24. The depression evaluated by RCADS correlated better with the PCS-C (0.43; *p* = 0.03) than with the PCS-P (0.3; *p* = 0.15). The FDI correlated strongly with RCADS depression (0.7, *p* =  < 0.001), moderately with PCS-P (0.34, *p* = 0.1), and not at all with PCS-C (0.05; *p* = 0.8) (Table 4).

**Table 4:** Correlation of daytime HRV data with psychological measures in healthy control and DGBI groups

	RMSSD			lf HRV			hf HRV			lf/hf		
	HC	DGBI	*p*-Value	HC	DGBI	*p*-Value	HC	DGBI	*p*-Value	HC	DGBI	*p*-Value
PCS-C	−0.59	−0.20	0.28	−0.51	0.05	0.17	−0.32	−0.33	0.97	−0.17	0.31	NS
PCS-P	−0.09	−0.21	0.78	−0.08	−0.23	0.73	−0.42	−0.34	0.83	0.31	0.28	NS
RCADS d	−0.54	−0.27	0.46	−0.41	−0.39	0.95	−0.49	−0.24	0.51	0.23	−0.17	NS
RCADS a	−0.72	−0.02	0.04	−0.66	0.07	0.05	−0.11	0.03	0.76	−0.39	−0.06	NS
RCADS a and d	−0.78	−0.16	0.04	−0.65	−0.14	0.14	−0.26	−0.13	0.77	−0.26	−0.07	NS
TSCC PTSD	−0.36	−0.35	0.98	−0.28	−0.23	0.89	0.00	−0.43	0.30	−0.23	0.25	NS
FDI	−0.03	−0.31	0.52	0.26	−0.46	0.09	−0.23	−0.29	0.90	0.34	−0.16	NS

*RCADS* d RCADS depression, *RCADS a* RCADS anxiety, *RCADS a and d* RCADS total anxiety and depression, *HC* healthy control, *DGBI* disorders of gut–brain interaction, *lf HRV*, low-frequency HRV, *hf HRV* high-frequency HRV

## Discussion

Summarizing the primary conclusions: (1) the findings match our prior work, showing lower vagal modulation in adolescents with DGBI compared with healthy subjects [2]; (2) in contrast to this prior report, the current DGBI cohort matched the healthy subjects in pain, catastrophizing, depression, anxiety, post-traumatic stress, and function levels presumably owing to active on-going management, suggesting that the HRV reduction was due to the DGBI itself rather than accompanying mood or pain issues; (3) this reduction in cardio-vagal modulation in the DGBI group occurred in both daytime and nighttime; (4) the DGBI group exhibited less nocturnal rise in RMSSD compared with healthy subjects; (5) cardiovagal modulation correlated inversely with catastrophizing in both child and parent, as well as depression and function in the child; (6) nocturnal HRV demonstrated a stronger inverse correlation than daytime HRV with parental catastrophizing and FDI (Table 5).

**Table 5:** Correlation of nighttime HRV data with psychological measures in healthy control and DGBI groups

	RMSSD			lf HRV			hf HRV			lf/hf		
	HC	DGBI	*p*-Value	HC	DGBI	*p*-Value	HC	DGBI	*p*-Value	HC	DGBI	*p*-Value
PCS-C	0.01	−0.06	0.88	−0.34	0.17	0.24	−0.08	−0.25	0.70	−0.12	0.58	NS
PCS-P	−0.02	−0.47	0.26	−0.07	−0.76	0.04	−0.15	−0.45	0.46	0.27	0.01	NS
RCADS d	0.25	−0.34	0.17	−0.10	0.01	0.80	0.14	−0.39	0.21	−0.31	0.49	0.05
RCADS a	−0.04	0.35	0.37	−0.38	0.20	0.17	−0.13	0.18	0.48	−0.13	0.38	NS
RCADS a and d	0.05	−0.01	0.90	−0.32	0.17	0.25	−0.05	−0.15	0.82	−0.20	0.54	Trend 0.07
TSCC PTSD	0.14	−0.34	0.25	−0.13	0.26	0.37	0.20	−0.49	0.09	−0.39	0.75	0.01
FDI	0.42	−0.45	0.03	0.40	−0.21	0.15	0.30	−0.43	0.08	−0.05	0.19	NS

*RCADS d* RCADS depression, *RCADS a* RCADS anxiety, *RCADS a and d* RCADS total anxiety and depression, *HC* healthy control, *DGBI* disorders of gut–brain interaction, *lf HRV* low-frequency HRV, *hf HRV* high-frequency HRV

Reduced vagal modulation has been reported in many pain conditions, including DGBI, though the few pediatric studies available show conflicting results. The results reported here corroborate our prior studies [2] in a different cohort with a different design where subjects were supine and then stood up. Both studies demonstrated that vagal modulation is significantly lower in the DGBI group. Importantly, since the DGBI group was doing well enough to have comparable FDI and psychological profiles as the HC, this suggests that differences in vagal modulation are not secondary to these associated factors but to some aspect intrinsic to the presence of a DGBI. We were surprised by this finding, particularly given that we found the expected inverse correlations between HRV and depression, and function and catastrophizing across the entire population.

Potential explanations for this important finding include: (1) the intrinsic nature of a DGBI reflects a dysregulation of brain–gut communication, as the name implies, based on vagal signaling, with abnormal vagal signaling extending to cardiac control and perhaps other end-organs if they were investigated; (2) the central neural circuitry behind a DGBI represents a broader shift in brain function to “survival” mode, which impairs vagal outflow in general; (3) afferent feedback from functional gut dysregulation entrains dysregulation in other organs. These possibilities could be sorted by directionally blocking vagal traffic in animal models of DGBI or examining the effect of vagal maneuvers in human subjects.

This study extends prior findings to nocturnal HRV. During the night, healthy subjects experienced a decrease in blood pressure and heart rate [7]. Both groups showed an expected increase in vagal modulation at night, but with significant attenuation in the DGBI group. Thus, the DGBI group showed two separate abnormalities: an overall reduction in cardiovagal modulation at night and a blunting in the expected increase in vagal modulation, even given the baseline reduction. In healthy normotensive young adults, blunted heart rate dipping at night was present in subjects with poor sleep efficiency, independent of total sleeping time [9]. This may have contributed to our findings, given the known sleep disruption in this population.

Less surprising findings included the inverse correlation of vagal modulation with function (based on the Functional Disability Inventory), implying that higher disability is associated with poorer vagal modulation as observed in several other disorders, for example, cervical pain [10].

In the same vein, catastrophizing in both children and parents correlated inversely with the child’s vagal modulation. Children often rely on their parents for emotional regulation, as parents play a key role in shaping the emotional regulation of the children [11], perhaps illuminating why catastrophizing in the children also correlated strongly with depression. In turn, the strong dependence of emotional regulation on vagal modulation [12] would explain why HRV is reduced when catastrophizing is high.

It is important to mention both the limitations and strengths of this study. Our findings show an association between low HRV and DGBI and demonstrate a characteristic but do not provide any evidence toward causality in either direction. Nighttime HRV is somewhat generic since we could not determine specific sleep stage HRV, though this requires a more complex setup with more sophisticated equipment. Some of the associations are weak, though still statistically significant. Medications could not be stopped for the 4–5 days required to minimize any autonomic influence on HRV (that condition would have significantly challenged recruitment). However, our previous publication using a very short HRV recording [13] showed similar findings when all medications that influence autonomic function were stopped for five half-lives. Our study also includes mainly female participants because our patients at the clinic are 90% female patients, matching the known sex distribution of DGBI in adolescents. This preponderance of female participants might have affected the result of some of the questionnaires when compared with the HC, though our prior work showed similar function and catastrophizing levels in adolescent girls and boys. Although girls tend to show more depression than boys [14], we did not find this difference in our cohort, probably limited by the asymmetry of the two populations.

The strengths include a solid cohort with 27 subjects and a good match of psychiatric and functional assessments across the two groups. This is the first study to report that the HRV differences remain even when mood and function are comparable. These findings are important and may lend credence to the emerging use of transcutaneous vagal stimulation in the treatment of DGBI. Vagal stimulation improves gastrointestinal symptoms and motility [15]. Future research should determine whether vagal stimulation requires improved vagal modulation to be effective, and whether inflammatory markers improve through the vagal anti-inflammatory pathway. Finally, understanding the underlying brain mechanisms would widen therapeutic approaches.

We conclude that HRV was reduced in a cohort of patients with DGBI and that this is not explained by the most important factors known to reduce HRV that we were able to assess such as depression, catastrophizing, or history of psychological trauma. Other factors influence HRV but were not assessed. However, impaired cardiovagal modulation may be an intrinsic characteristic of DGBI, though larger studies with repetitive vagal measurements are needed to confirm this hypothesis.

## Data Availability

The data are available upon reasonable request for release to a faculty of an academic institution in good standing for 2 years.
